# Evaluating the external validity of an artificial intelligence-based mobile support app for caregiving relatives by an online expert survey

**DOI:** 10.1186/s12911-026-03407-2

**Published:** 2026-02-27

**Authors:** Dominik Wolff, Michael Marschollek

**Affiliations:** Peter L. Reichertz Institute for Medical Informatics of TU Braunschweig and Hannover Medical School, Hannover Medical School, Hannover, Germany

**Keywords:** Evaluation, External validity, Knowledge-based system, Expert system, Nursing, Informal care, Health literacy, Mobile health

## Abstract

**Background:**

Mobile Care Backup is a support app for family caregivers that provides textual information on topics personalized to their specific care situation. Personalization is performed by an artificial intelligence-based expert system. Here, we present the evaluation of the expert system’s validity with project external nursing experts. Furthermore, we discuss the general limitations of an online survey as an evaluation methodology for expert systems.

**Methods:**

This study was conducted as an online survey in German and English. A total of nine experts, all of whom were female and had extensive (outpatient) care experience, were included. The participants were presented with descriptions of multiple fictitious family caregivers and the system’s personalized list of topics. They were then asked to rate the appropriateness on a five-item Likert scale and suggest additional topics. The collected data was analyzed descriptively to investigate whether MoCaB‘s topic recommendation strategy aligns with project external experts. For deviating topic sequences, the consensus of the experts was verified by pairwise rank correlation using Spearman’s Rho. Additional suggested topics were checked to see if they were part of the system but not provided (false negatives).

**Results:**

In the 495 submitted ratings, participants rated the suggested topics‘ appropriateness relatively high, with an average rating of 4.4 and a median of 5. This indicates that participants consider most of the recommended topics important for the fictitious family caregiver. The system‘s personalization performance was high (precision of 0.965 and recall of 0.986). Overall, the experts are unanimous. There is no unique alternative sequence regarding the rare cases of disagreement with the system in the ordering of topics.

**Conclusions:**

The MoCaB system’s external validity is high, and isolated inconsistencies will be resolved in the project group. Using an online survey to evaluate the system’s validity with external experts is complex and time-consuming. Participants need a very high degree of competence, as they must infer from the title to the content. Nevertheless, it is an essential step in the evaluation process of expert systems and, if carried out correctly, can identify weak spots and further improve the expert system.

**Supplementary Information:**

The online version contains supplementary material available at 10.1186/s12911-026-03407-2.

## Background

Population’s low health literacy is one key issue in worldwide healthcare systems [1, 2]. Low health literacy is associated with worse physical mobility [3], higher hospitalization rates [4], and even higher mortality rates [5, 6]. Over half of Germany‘s population has difficulties finding, understanding, assessing, or applying relevant healthcare information [7]. On the other hand, the population is a crucial part of the nursing system as informal caregivers who care for relatives such as spouses or parents. In Germany, 6% (5 mio.) of the population required care in 2021, with more than half of that (3.11 mio.) being exclusively cared for by relatives at home [8]. The mobile application *Mobile Care Backup (MoCaB)* [9, 10] aims to increase health literacy, especially the nursing literacy of caregiving relatives, by providing them with information on care-related topics personalized to their specific care situation and with basic nursing information suitable for every caregiver. Therefore, the system selects predefined texts from an expert curated corpus of 86 topics that are regarded as helpful for the specific user. Overall, the 86 topics are structured in four main categories: *legal and financial benefits*, *Day-to-day care*, *external support services* and *my role as a caregiving relative*. Single texts describe information belonging to specific topics such as *leisure activities after a stroke* and *coping with stress*. These texts are structured in form of predefined dialogs, where the user can ask topic-related predefined questions and the MoCaB system answers them. By chatting with an artificial expert in nursing and homecare as well as regularly being provided with trustworthy information, the health literacy and especially nursing literacy of the user should be increased. The personalization of the topics to the user’s care situation is implemented by an artificial intelligence system comprising multiple nursing experts’ explicit and tacit knowledge [11]. Overall, the expert topics of the MoCaB system are matched with the profile of the user to identify relevant topics for him/her. The personalization engine consists of two main components: an ontology and a scoring-based approach which is inspired by fuzzy-logic. In the ontology, nursing experts formalized rules which match topics with seven different properties of a family caregiver‘s profile such as the disease of the relative who is being cared for or whether both live in the same apartment. With these rules, topics are selected that are relevant for the user. A person who cares for a demented relative, for example, will be provided with medical information about dementia and the course of the disease but not with medical information regarding caring for someone after a stroke. On the other hand, a score for each possible topic is calculated providing a quantitative statement about the importance of topics. Here, the experts‘tacit knowledge which cannot be formalized in an ontology is used. For every topic, the experts assigned weights to the items of two assessment tools, which indicate how important it is that the item is true for the caregiver to provide the topic to him or her. Here, the caregiver burden index and a German national scale which assesses the independence, as a measure of support needed, of a person being cared for are used, enabling the system to infer from support needs and burdens to relevant information which then is provided by the system. By combining the experts‘weights and the caregiver’s profile regarding the two assessments, a score between zero and one is calculated for each topic by a mathematical function. The score indicates the relevance of an individual topic for the caregiver. Topics with scores larger than 0.3 are regarded as relevant while topics with lower scores will not be shown to the user. [11] Ultimately, the two approaches, the ontological and the scoring-based one, are combined by the intersection of topics identified as relevant and are presented to the user in descending score order. For a comprehensive overview of the system in German, please refer to [12, 13]. Before such a system can be used in a real-world setting, an extensive evaluation must be carried out to ensure that the system is both functional and helpful.

Standard approaches for evaluating knowledge-based systems, such as *Clarke et al.* [14] or *O’Keefe and O’Leary* [15], propose a multi-stepped iterative procedure. In the first step, severe errors in the general system are detected by prototype testing. This is followed by an evaluation of the system’s validity. Here, it is checked whether the knowledge-based components of the system, precisely the knowledge base and the inference algorithm, behave as intended by the experts. Sometimes, this step is further divided into internal and external validity, referring to evaluation against the experts who provided their knowledge (internal) and independent experts (external). The assessment of internal validity ensures that the knowledge-based component is functional, while the external validity evaluation confirms the systems‘generalizability. In the fourth step, a field study in the target group checks whether the system as a whole is functional. The final step is to assess the consequences of widespread system deployment.

The *MoCaB* system already passed the first two evaluation stages. The prototype testing comprised think-alouds and evaluation of the provided texts‘comprehensibility with ten participants, and a usability test of one week with six participants [16]. Afterward, the internal validity of MoCaB’s personalization engine, which comprises the knowledge base and the inference algorithm, were extensively examined and uncovered issues were resolved afterward [17].

Here, we present the evaluation of MoCaB’s external validity in the form of an online survey with nursing experts as the target group. By presenting experts who were not involved in the development stage with fictitious caregiver case studies and how the system would behave in these cases, i.e. which topics would be recommended, we assessed if the personalization of the MoCaB system works as intended from a project external expert view. Further, we discuss the feasibility of such an evaluation approach for topic recommendation systems in medical domains.

## Methods

**Fig. 1:**
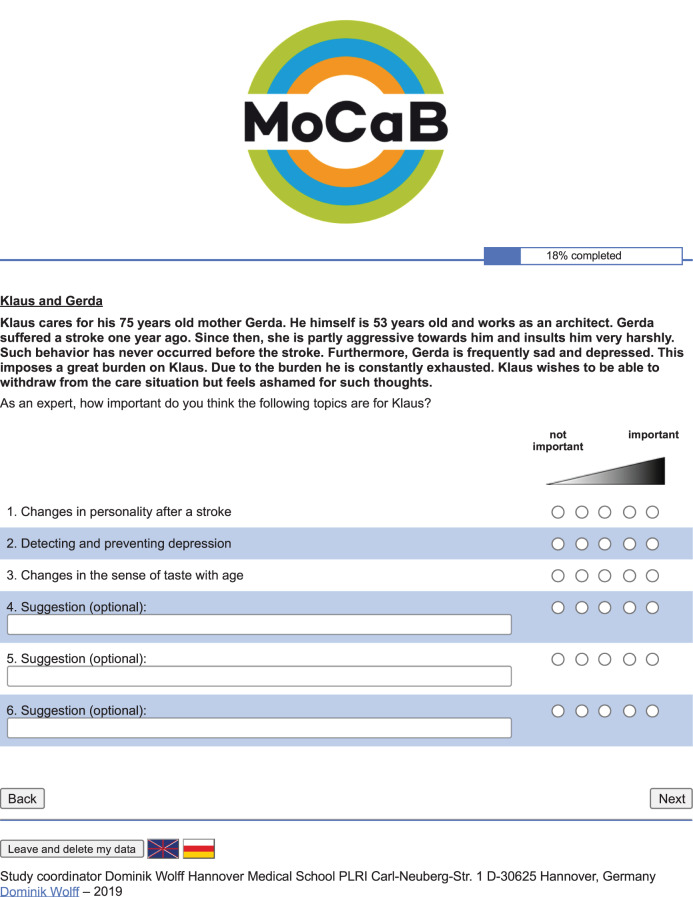
Screenshot of the online survey for the case study of a person caring for their relative after a stroke, which is based on the topic ‚Changes in personality after a stroke‘

To assess the external validity of MoCaB’s artificial intelligence component, a dynamic evaluation was performed with the nursing experts who were not part of the development. In contrast to static evaluations, where automated system checks are performed, the system is run for one or multiple scenarios, and experts then review the system’s output. In the case of MoCaB, fictitious case studies of caregiving relatives and a list of topics personalized by the system for each case were created. The individual case studies are based on one specific topic each. The characteristics that a caregiving relative must satisfy to be presented with the specific topic are used to create a description of the case study and as input for MoCaB’s topic recommendation system. In the example shown in Fig. 1, the topic *Changes in personality after a stroke* was used to create the case study. Therefore, the textual description presents the case of a fictitious caregiving relative who satisfies all features necessary for *Changes in personality after a stroke* to be relevant, such as caring for someone suffering from a stroke who is partly aggressive towards the caregiver. Following the textual description, the titles of topics identified as important for this fictitious caregiver are presented in order of decreasing calculated importance. Basing the case studies on one specific topic leads to more unrealistic descriptions of the care situation but improves the overall analytical insight of the study since misconceptions can be traced more easily. Besides, a case study of a realistic caregiving relative would be much more burdened and, therefore, would be presented with more information. Such realistic cases are not suitable for assessing the level of personalization. In total, eight case studies were created, with a wide range of topics as a basis (see Additional File 1). In these eight fictitious case studies, 55 topics are suggested by the system. For reporting, the CROSS checklist for Reporting of Survey Studies [18] is applied (also see Additional File 2). The study was not registered in a dedicated study register due to varied reasons. First, the study does not contain direct intervention and is comparable to a consensus study. Second, data was collected anonymously. Third, the direct results only apply to the MoCaB system, and the cohort is not representative of a population.

After giving informed consent, sociodemographic data were collected at the beginning of the survey including *profession*, *years of experience*, *years of experience in outpatient care*, *highest degree*, *gender* as well as the *working location (country)*. Following, the experts were presented with the cases and asked to rate the importance of each topic for the presented caregiving relative on a five-point Likert scale, ranging from zero (not important) to five (highly important). Additionally, the experts could specify up to three more topics, which would be relevant for this case but were not listed (see Fig. 1). In case of not reviewing one or more of the given topics, an automated question was asked whether the expert would like to answer or to proceed without giving information. The study was conducted as an online survey with a German and an English version. The survey was implemented using SoCi Survey [19] running on a server of Hannover Medical School. After evaluating the survey in a technical check with three persons as well as a usability study with five participants and resolving uncovered issues, the study was carried out from 08.09.2019 to 15.01.2021.

The study’s target group includes people with a degree and experience in nursing, preferably in outpatient care. The participants were recruited via snowball sampling [20]. A link to the survey was sent to representatives of the *International Medical Informatics Association (IMIA)* working groups *Nursing Informatics*, *Nursing Informatics Education* und *Student and Emerging Professionals Special Interest Group* as well as the *European Federation for Medical Informatics (EFMI)* working group *Nursing Informatics*. Furthermore, representatives of the respective departments of *Aalborg University* (Denmark), *University of Turku* (Finland), *Columbia University* (United States of America), *Carl von Ossietzky University of Oldenburg* (Germany), *Ostfalia University of Applied Sciences* (Germany) and *Benemérita Universidad Autónoma de Puebla* (Mexico) as well as the *Johanniter-Unfall-Hilfe e.V.,* a large German voluntary humanitarian organization, and outpatient nursing staff and nursing service managers from Germany and Austria asked to participate and to forward the survey to the target group. Over 50 people were contacted directly.

In total, the questionnaire was accessed 72 times. In most cases, the survey was aborted before completion. For ten participants, complete data is present. One of the ten participants was excluded since the sociodemographic data was not interpretable. The remaining nine participants can be characterized as experienced outpatient and geriatric nurses, and outpatient care managers, who are all female, with at least 10 years of professional experience. Two participants possess a university degree. Eight participants work in Germany, and one participant works in Mexico (see Table 1).

**Table 1:** Sociodemographic description of the study’s population. Answers with identical meanings have been standardized in wording and spelling. German answers were translated into English

ID	profession	highest degree	years of experience	years of experience in outpatient care	gender	working location (country)
1	nursing	registered nurse	20	10	female	Germany
2	nursing	outpatient care management	30	23	female	Germany
3	nursing/outpatient care management	general higher education entrance qualification	12	4	female	Germany
4	nursing	general higher education entrance qualification	31	2	female	Germany
5	nursing	Master	10	6	female	Mexico
6	outpatient care management	secondary compulsory education	33	20	female	Germany
7	nursing	secondary compulsory education	40	25	female	Germany
8	registered nurse	Bachelor	13	4	female	Germany
9	nursing	secondary compulsory education	21	0	female	Germany

The collected data was analyzed descriptively to investigate whether MoCaB‘s topic recommendation strategy is in line with project external experts. Regarding the survey, the following three aspects were of interest

- topics which are identified by the system as important but not by the external experts
- the ordering of important topics
- additional suggested topics that were missing.

For the first aspect, ratings below the 2σ-interval surrounding the mean of all collected ratings were regarded as stating that the topic is unimportant. Analysis of the topic’s ordering was based on the five Likert scale items. In contemplation of being interested in major mistakes and not minor disagreements between experts, a minimum difference of two on the Likert scale was used as criterion. This means, from the point of view of the ordering stated in the case report, that if, for example, the first topic is rated with a four and the second topic is rated with a five, this will be regarded as a minor disagreement. But in case the first topic is rated with a three or less and the second is rated with a five, a major difference in order is present, which needs to be investigated. To validate if the individual participants who disagreed with the system’s ordering of topics all agreed on the same ordering, pairwise rank correlation was calculated using *Spearman’s Rho.* The results are plotted as heatmaps. For the additionally suggested topics, it is checked whether MoCaB includes these or similar topics. In the survey, only the topics personalized by the system are listed, which would be broadened by basic topics suitable for every caregiving relative in a realistic setting. In order to evaluate the topics additionally suggested by the surveyed experts, the suggestions were classified into four groups: 1) Topics that are already included in the system’s personalized topic suggestions for the fictitious caregiver. This means that the participants suggested information that are already identified as relevant by MoCaB but maybe under a slightly different name or are part of one of the topics identified as relevant for the fictitious caregiver. Examples and reasons why this is possible can be found below in the discussion section. 2) Topics that are part of MoCaB’s basic information about caregiving, which are relevant for all users, and therefore not personalized and hence not listed in the topic suggestions for the fictitious caregiver. 3) Topics that are part of the MoCaB system but are not listed for the fictious caregiver. This means that the system missed these relevant topics in the personalization and therefore additional suggestions falling in this categegory are false-negatives. 4) Topics that are not included in the MoCaB system at all. These suggestions may be important for the user, but are not part of MoCaB’s topic corpus, which is currently limited to 86 topics. This category of additional suggestions does not provide any information about the personalization quality of the system, which is the focus of this study, but it gives a good indication for future expansion of the corpus.

Especially critical are topics that are part of the MoCaB system but are not listed for the fictitious caregiver. While the experts surveyed find them important for the fictitious caregiver, the system does not. For these topics, it must be checked if there is an error in the system‘s knowledge base.

Finally, the system‘s precision and recall were calculated. Again, ratings below the 2σ-intervall surrounding the mean of all collected ratings were regarded as false-positives, while additional suggestions which are part of the MoCaB system, but are not listed for the fictitious caregiver are regarded as false-negatives. All other possibilities are regarded as correct-classifications.

## Results

The mean of all 495 participants‘ratings of the 55 recommended topics was 4.4, and the median was 5 on a Likert scale from one to five, while the standard deviation was 0.843. Overall, the participants consider the majority of the recommended topics to be important. The standard deviation of ratings for each of the 55 individual topics ranged between 0.0 and 1.414. For 45 topics, it was less than one. This indicates that the participants, in general, had a common opinion on the system’s recommended topics.

The lower border of the 2σ-interval was 2.714 scale points, resulting in ratings with one or two scale points being viewed as false-positives. In total, eighteen ratings from five participants fall in this category. Fifteen of those come from two participants (ID 3: ten and ID 6: five). Most of the eighteen topics were rated with two or less scale points by one or two participants. Only one topic was considered unimportant by three participants. Two other participants rated the same topic in the same use case with three, three participants with four, and one participant with five Likert scale points. Overall, false-positives are exceptional.

**Fig. 2:**
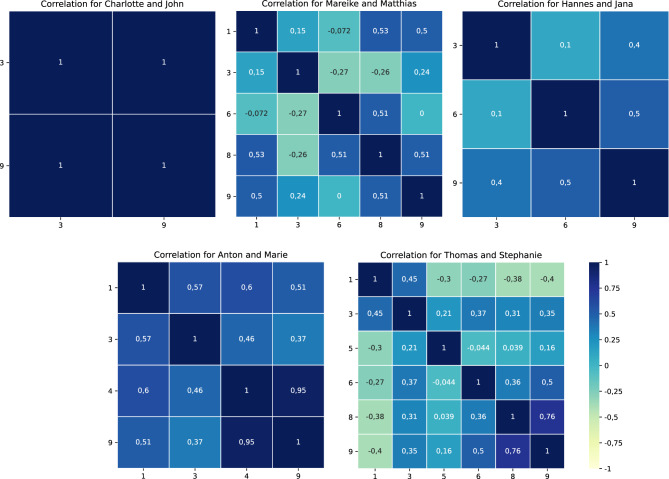
Pairwise Spearman rank correlation for orderings where the participants disagreed with the system [13]. A higher value (blue) indicates that two participants agree on a specific ordering with 1 representing a perfect match in ordering, while a lower value (yellow) indicates that the orders are contradictory with −1 representing the exact opposite order. Zero indicates that no correlation, neither positive nor negative, was found

For six fictitious case studies, significant differences in the system’s ordering and ordering based on participants’ feedback occurred (see Table 2). Here, participants with IDs 3 and 9 are the most prominent. Especially critical are the case studies *Mareike and Matthias,* and *Thomas and Stephanie*, where the order differs for more than half of the participants. Figure 2 shows the Spearman rank correlation for the five case studies where more than one participant disagreed with the system’s ordering. The higher the value, the higher the agreement between participants. The case study *Charlotte and John* showed a remarkably high correlation between the participants with ID 3 and 9. For this case study, the MoCaB system only recommended two topics, and therefore, only two orderings are possible. So, either a participant agreed with the system’s recommended ordering, or the participant voted for the only other possibility leading to a perfect correlation between all participants not agreeing with the system’s ordering. All participants show (strong) pairwise positive rank correlation for the case study *Anton and Marie*. The pairwise correlation is low or even negative for the other three use cases, with single exceptions. For the especially critical case studies, where more than half of the participants disagreed with the system’s ordering of topics, there seems to be a correlation between the number of topics and the number of participants disagreeing with the system’s ordering. The more topics were selected to be important by the system for the case study, the more participants disagreed. Except for the case study *Anton and Marie,* the participants did not agree on a specific ordering in the cases of significant differences with the system’s suggested ordering.

**Table 2:** Overview of significant differences in participant’s and system’s ordering grouped by case studies

case study	participant ID
Klaus and Gerda	/
Charlotte and John	3, 9
Mareike and Matthias	1, 3, 6, 8, 9
Hannes and Jana	3, 6, 9
Alina and Charlie	/
Anton and Marie	1, 3, 4, 9
Dieter and Helga	3
Thomas and Stephanie	1, 3, 5, 6, 8, 9

The participants suggested 40 additional topics for the eight case studies. For further analysis, the suggested topic *aggressions due to dementia* is excluded since the use case describes a care setting after a stroke. Seven of the suggestions are already included in MoCaB’s recommended topics. Seventeen further suggestions are included in the basic knowledge topics, which are not part of this evaluation. Eight suggested topics are not included in the MoCaB system, while seven are present in the system but not in the recommended topics for the specific case study (see Table 3).

**Table 3:** Overview of additional suggested topics that are already included in MoCaB but are missing for the fictitious case study

suggested topic	case study	participant ID	included in
leisure activities after a stroke	Klaus and Gerda	1	leisure activities for people suffering from a stroke
biography work	Charlotte and John	1	How to use biography work?
leisure activities for dementia patients	Charlotte and John	1	leisure activities for dementia patients
possible problems with swallowing beverages and beverage thickening	Alina and Charlie	4	swallowing and swallowing disorders
leisure activities for dementia patients	Anton and Marie	1	leisure activities for dementia patients
continence management	Dieter and Helga	4	structuring toilet visits
protective utensils, diapers, pads – which to use	Dieter and Helga	9	handling of incontinence material

Considering the mentioned definitions of false-positives, false-negatives and correct-classifications, the systems showed a precision of 0.965 and a recall of 0.986 in this survey. A high external validity was shown for the MoCaB system.

## Discussion

Regarding the MoCaB system, high individualization performance was shown in an experienced cohort of external nursing experts. Overall, the participants were unanimous, and negative evaluations were the exception, with 18 out of 495 submitted ratings. For topic orderings deviating from the system, participants disagreed on the topics‘sequence. The more topics were suggested for a persona by the system, the more diverse the experts’ orders. An exception is the persona *Anton and Marie*, where strong correlations for four participants were shown. Here, we were able to identify a potential error in the system. The systems ordering does not fit the experts ordering to a large extend. There are two potential reasons for this. First, discrepancies in the knowledge base, i.e. the project internal experts’ weighting for the topics in question, could lead to different orderings as intended. Since the participants still regarded these topics as relevant for the fictitious caregiver, we expect only minor changes in the knowledge base for this case. Second, the cause of this difference in ordering could be based on the artificial nature in creating the fictitious caregivers. By choosing one specific topic as the profile basis for the creation of each fictitious caregive, these case studies, and especially the topics ordering, can differ from realistic caregivers. We will further inspect this issue together with the project’s internal experts. For this, we will investigate both plausible causes. For the first, we will assess the persona and the knowledge base in regard of potential errors with the nursing experts, while for the second possible cause, we will alter the profile of the persona *Anton and Marie*, to be more realistic and reassess the results with both project internal and external experts. Nevertheless, this highlights the importance of this evaluation step when implementing a knowledge-based system. Regarding topics additionally suggested by the participants, the majority is part of the basic nursing information shown to every caregiver, which is not part of the individualization and hence is not included in this evaluation. Another part of the suggested topics is already present in the list of topics for the persona. This highlights a drawback of the evaluation approach itself. Since only the heading of each topic was displayed, the participants had to imagine the content shown to the caregivers based on these headings. Therefore, such inaccuracies are possible. For example, the suggested topics *appropriate sleep medication* and *changes in the day-night rhythm due to dementia* are part of MoCaB’s topic *How does Sleep change with age?*. Presenting the participants with the complete content of each topic, on the other hand, would make the evaluation impossible in terms of time and hold the risk of shifting the feedback to content quality instead of individualization performance. The content quality was assessed in a prior evaluation [16]. Still, seven suggested topics are part of MoCaB but were not suggested for the corresponding personas. A plausible reason could be how we created the fictitious characters. They are each based on the profile items used to personalize one topic, which can lead to a very isolated and unrealistic burden and care situation. A realistic fictitious character would show additional burden in similar profile items, which are missing for these non-realistic ones. Nonetheless, the expert knowledge on these seven topics needs internal reassessment. In particular, the topic of *leisure (dementia)*, which was not proposed in the system sequence for two different personas, requires further inspection. The prior mentioned suggestion of the topic *aggressions due to dementia*, which was excluded since the use case described a care setting after a stroke, could be of interest to a realistic caregiver in such a setting as dementia can be a consequence of a stroke [21].

Nonetheless, there are factors limiting the positive evaluation results. First, despite major recruitment efforts, the small number of participants limits the results. Both the click rate as well as the completion rate are low. Regarding the click rate, potential reasons are missing incentives, lack of time and non-optimal recruitment. Besides supporting science, no incentive such as financial compensation which would be quite common for participating in a study was given. Financial incentives are known to have a strong positive influence on the participation rate [22, 23]. Further, the target group of the survey consists of highly burdened experts, in terms of time and number of requests. Despite directly contacting over 50 persons who should function as multipliers for the snowball sampling, only 72 clicks were generated. This highlights issues in the recruitment of participants. Only one initial and a follow-up message were sent to them. It is possible that the follow-up did not reach the persons contacted by the multipliers. A more personal additional contact, for example via a phone call, could potentially increase the participation rate. For the low completion rate, potential factors are the ease of completion and the topic knowledge [23], which influence each other. The task itself is, as discussed below, cognitively challenging as the participants need to empathize with multiple fictitious caregivers and infer the caregivers’ potential knowledge gaps. Even more, some participants contacted us stating that their knowledge level was not fitting the task as they have not worked practically for too long. Here, focusing on experts who are still practicing and a good introduction to the system could help. It could have been helpful to give the experts access to the MoCaB system prior to sending the survey, but this was not feasible since the distribution of the MoCaB app was not accessible for the public and relied on direct installation to the phone at that time point. Regarding these two problems, a possibility to show the complete MoCaB texts if desired could reduce the complexity of the task. Second, all participants were female, making it impossible to analyze gender aspects. Third, the cohort is characterized by a substantial proportion of German participants. This is only a minor limitation since MoCaB was developed for the German nursing system and includes topics focusing on information specific to this setting. When transferred to other countries, the system‘s topics, as well as knowledge base, would need inspection and adjustments. Fourth, it is highly likely that the meaningfulness of the false-negative rate is limited. As the subjects cannot know the complete set of topics covered by the MoCaB system, their additional suggestions are likely not complete, having a direct influence on the false-negative rate. This could be tackled in the future by having an additional drop-down menu containing all topics included in the system for the additional suggestion space, which would further complicate the evaluation task. Fifth, displaying the topics by decreasing importance to the participants could introduce a bias in their rating behavior. Since not only the overall importance of topics but also the ordering of topics were evaluated, the only way to detect such a bias would be to subdivide the participants into two groups and perform A/B testing. To one group orderless topics would be displayed, while the other group would remain as described above. Unfortunately, recruitment problems made this impossible.

In regard of the recently advancing large language models, we are expecting that such systems as MoCaB will greatly benefit from their language processing capabilities. They will improve user interaction and reduce the need for expert curated texts. Especially, we see high potential in retrieval augmented generation, which allows higher accuracy and specialization to a specific topic like home care. For such systems, the here proposed evaluation methodology has drawbacks. The preparation of fictitious case studies and the corresponding output of the large language model would have a higher complexity. Granting the participants access to the model would negate this point, but it would be necessary to track all user interactions resulting in an unstructured dataset which’s analysis is more complex and not that suitable for an evaluation study.

In general, the evaluation of an expert system with project external experts is an important step in the implementation of such a system to ensure its objectivity [14, 24]. However, this evaluation step is rarely incorporated and implemented during development. Many expert systems do not reach an application in the real world and often get stalled after the internal validity evaluation, even when producing promising results. Potential reasons are lacking standardization of the external validity evaluation step and the high recruitment effort. The target group of recruiting typically consists of a small group of experts which size varies with the system’s field of application. Introducing financial compensation could help to address the latter. Nonetheless, the expertise needed to participate in the evaluation of a topic recommendation system is immense. First, participants need knowledge of the field, here, informal care. Second, they must familiarize themselves with several fictitious characters in a short time. And most important, they must infer the potential content of the proposed topic from the title, which is a cognitively complex task. Several experts who were asked to participate returned feedback that they could not complete this task. Altogether, this highlights a strong limitation of the proposed method. Furthermore, it is essential to incorporate at least one realistic fictitious character in the evaluation as described above. The discrepancies identified must then be reviewed again with the experts involved in development.

While the evaluation was positive for the MoCaB system, the direct results only apply for MoCaB and are not generalizable to other applications. Still, future researchers will benefit from our lessons learned. First, do not underestimate the recruitment effort. Giving financial incentives as well as personal contact can increase the participation rate. Before you start, carefully decide on the survey’s target group. For practice-oriented systems, it may make sense to focus on persons who are still practicing instead of very academic experts. Second, include a possibility of having access to all possible content to reduce false negatives. Third, include realistic case scenarios, which help to evaluate the readiness for a field study. Fourth, provide the participants with direct access to the developed system prior to the survey, if possible, or provide a detailed introduction to the system at the start of the survey.

## Conclusions

The methodology of an online survey for the validity evaluation with external experts is complex and time-consuming. Furthermore, participants need a remarkably high degree of competence. Nevertheless, it is a crucial step in the evaluation process, which, if carried out correctly, can identify weak spots and further improve the expert system. For the MoCaB system, an overall high external validity was shown. Discrepancies found will be examined and dealt with within the project team. After ensuring that the system works as intended, the next step is to assess the usefulness of the MoCaB by a prospective evaluation of the functionality of the entire MoCaB system with caregiving relatives in a real-world setting. Here, it will be assessed if providing family caregivers with personalized information increases their health literacy while not increasing burden. To this end, the app will be made available to family caregivers on their own or borrowed mobile devices for at least 4 weeks in a home care setting. A mixed methods approach will be applied, using semi-structured interviews to assess content aspects, usage aspects (like frequency, overall evaluation, recommendation, willingness to pay), technical aspects (like functionality and technical problems) and design aspects (like comprehensibility, number of daily suggested topics, structure, color design) together with digital data captured directly while using the app such as the topics suggested, if they were read as well as a user rating for each topic.

## Electronic supplementary material

Below is the link to the electronic supplementary material.

Supplementary material 1

Supplementary Material 2

## Data Availability

The datasets supporting the conclusions of this article are available from the corresponding author upon reasonable request.
