# Supplementary material for: Evaluating the external validity of an artificial intelligence-based mobile support app for caregiving relatives by an online expert survey
Source: BMC Med Inform Decis Mak. 2026 Feb 27;26:58. doi: 10.1186/s12911-026-03407-2 (PMC12952002; doi:10.1186/s12911-026-03407-2)
Supplement: Supplementary file 1 — Supplementary material 1 [file 12911_2026_3407_MOESM1_ESM.pdf]

## Screenshots of the English online survey

0% completed

Dear nursing experts,

we would like to ask you to participate in our survey on the project **Mobile Care Backup**. In the German federal ministry of education and research funded project “Mobile Care Backup (MoCaB)” (grant number 16SV7472) an automated educational system for caregiving relatives is developed. The information presented is personalized to the caregivers' and patients' needs. This personalization is based on the knowledge of four nursing experts. Aditionally to the personalized information, every caregiver is presented basic care information which is not personalized to her or his profile and thus does not appear in this survey.

With this survey, we would like to evaluate the matching of provided information and the user's profile with experts from outside the project in order to improve the personalization. Therefore, you will be presented 8 fictitious caregivers consisting of a short profile description and a listing of titles of the topics provided individually for this caregiver. We would like to ask you to rate the importance of the topics (0 = not important, 5 = important) for the provided profile. If you have further ideas for topics that are important for this profile, please add them in the sections provided. **This survey will take 15-20 min.**

Thank you!

**Declaration of Consent**

**Your participation in the study is voluntary. You may terminate your participation in the study at any time without giving reasons. Information you enter will be transferred to servers within the Hannover Medical School in accordance with the data protection laws and processed. After completion of the study, your data will be stored for ten years in accordance with the guidelines of good scientific practice and irrevocably deleted at the end of the study.**

○ I agree with the fact that the data raised from me are stored anonymously on servers of the Hannover Medical School and processed for the purpose of evaluation. Consent to the collection and processing of the data is irrevocable, as no participant-related deletion can be carried out due to the anonymous form of the survey. I agree that my data will be deleted after completion or discontinuation of the survey after it has been stored for at least ten years.

Study coordinator
Dominik Wolff
Hannover Medical School
Peter L. Reichertz Institute for Medical Informatics
Carl-Neuberg-Str. 1
D-30625 Hannover, Germany
Dominik.Wolff@PLRI.de

Next

Leave and delete my data

9% completed

## 1. Personal Information

| | |
|---|---|
| Profession (i.e. Nursing): | |
| Highest educational qualification : | |
| Years of experience: | |
| Years of experience in ambulant care: | |
| Place of work (country): | |
| Gender: | [Please choose] |

Back

Next

Leave and delete my data

Study coordinator Dominik Wolff Hannover Medical School PLRI Carl-Neuberg-Str. 1 D-30625 Hannover, Germany

Dominik Wolff – 2019

18% completed

**Klaus and Gerda**

**Klaus cares for his 75 years old mother Gerda. He himself is 53 years old and works as an architect. Gerda suffered a stroke one year ago. Since then, she is partly aggressive towards him and insults him very harshly. Such behavior has never occurred before the stroke. Furthermore, Gerda is frequently sad and depressed. This imposes a great burden on Klaus. Due to the burden he is constantly exhausted. Klaus wishes to be able to withdraw from the care situation but feels ashamed for such thoughts.**

As an expert, how important do you think the following topics are for Klaus?

| | not important | | | | important |
|---|---|---|---|---|---|
| 1. Changes in personality after a stroke | ○ | ○ | ○ | ○ | ○ |
| 2. Detecting and preventing depression | ○ | ○ | ○ | ○ | ○ |
| 3. Changes in the sense of taste with age | ○ | ○ | ○ | ○ | ○ |
| 4. Suggestion (optional): | ○ | ○ | ○ | ○ | ○ |
| 5. Suggestion (optional): | ○ | ○ | ○ | ○ | ○ |
| 6. Suggestion (optional): | ○ | ○ | ○ | ○ | ○ |

Back Next

Leave and delete my data

Study coordinator Dominik Wolff Hannover Medical School PLRI Carl-Neuberg-Str. 1 D-30625 Hannover, Germany
Dominik Wolff – 2019

MoCaB

27% completed

**Charlotte and John**

**John (67) cares for his demented partner Charlotte. She is 70 years old. Charlotte needs help with dressing and other activities. She is overextended with everyday decisions, but concurrently cannot admit that she is dependent on help. This makes John sorrowful and enraged. When her children come to visit, John is embarrassed about the way Charlotte is dressed. This often leads to an argument.**

As an expert, how important do you think the following topics are for John?

| | not important | | | | important |
|---|---|---|---|---|---|
| 1. Clothing as an expression of individuality in people suffering from dementia | ○ | ○ | ○ | ○ | ○ |
| 2. Psychological changes of demented persons | ○ | ○ | ○ | ○ | ○ |
| 3. Suggestion (optional): | ○ | ○ | ○ | ○ | ○ |
| 4. Suggestion (optional): | ○ | ○ | ○ | ○ | ○ |
| 5. Suggestion (optional): | ○ | ○ | ○ | ○ | ○ |

Back

Next

Leave and delete my data

Study coordinator Dominik Wolff Hannover Medical School PLRI Carl-Neuberg-Str. 1 D-30625 Hannover, Germany
Dominik Wolff – 2019

36% completed

**Mareike and Matthias**

**Mareike is 60 years old and works as an office clerk. She takes care of her demented spouse Matthias. Mareike sleeps poorly due to Matthias' disorientation at night. Matthias is capable of changing his position in bed independently, which frequently awakens Mareike. She feels emotionally and physically exhausted due to the care.**

As an expert, how important do you think the following topics are for Mareike

| | not important | | | | important |
|---|---|---|---|---|---|
| 1. How does sleep behavior change with age? | ○ | ○ | ○ | ○ | ○ |
| 2. Sources of sleeping problems | ○ | ○ | ○ | ○ | ○ |
| 3. Suggestions for a restful sleep | ○ | ○ | ○ | ○ | ○ |
| 4. Physical activities for preserving health | ○ | ○ | ○ | ○ | ○ |
| 5. Coping with stress | ○ | ○ | ○ | ○ | ○ |
| 6. Dealing with nocturia | ○ | ○ | ○ | ○ | ○ |
| 7. Detecting and preventing depression | ○ | ○ | ○ | ○ | ○ |
| 8. self-conscious movements (Kinaesthetic) - definition and relief in care | ○ | ○ | ○ | ○ | ○ |
| 9. Exhaustion in caregiving relatives | ○ | ○ | ○ | ○ | ○ |
| 10. Suggestion (optional): | ○ | ○ | ○ | ○ | ○ |
| 11. Suggestion (optional): | ○ | ○ | ○ | ○ | ○ |
| 12. Suggestion (optional): | ○ | ○ | ○ | ○ | ○ |

Back Next

45% completed

**Hannes and Jana**

**The pensioner Hannes takes care of his partner Jana, who has been diagnosed with dementia two months ago. He does not get enough sleep since he must take care of Jana constantly, even at night. Jana is temporally and locally disoriented, especially in the evening, and frequently wanders aimlessly through the apartment.**

As an expert, how important do you think the following topics are for Hannes?

| | not important | | | | important |
|---|---|---|---|---|---|
| 1. Restlessness at sundown (sundowning syndrome) | ○ | ○ | ○ | ○ | ○ |
| 2. How does sleep behavior change with age? | ○ | ○ | ○ | ○ | ○ |
| 3. Suggestions for a restful sleep | ○ | ○ | ○ | ○ | ○ |
| 4. Sources of sleeping problems | ○ | ○ | ○ | ○ | ○ |
| 5. Dealing with the patient’s restlessness | ○ | ○ | ○ | ○ | ○ |
| 6. Dealing with nocturia | ○ | ○ | ○ | ○ | ○ |
| 7. What is a tendency to move? | ○ | ○ | ○ | ○ | ○ |
| 8. Wandering of demented persons | ○ | ○ | ○ | ○ | ○ |
| 9. Eat by walking – How to deal with restlessness while eating | ○ | ○ | ○ | ○ | ○ |
| 10. Suggestion (optional): | ○ | ○ | ○ | ○ | ○ |
| 11. Suggestion (optional): | ○ | ○ | ○ | ○ | ○ |
| 12. Suggestion (optional): | ○ | ○ | ○ | ○ | ○ |

Back Next

Leave and delete my data

55% completed

**<u>Alina and Charlie</u>**

**The 42 years old Charlie has the feeling that his mother is increasingly dependent on support due to problems in communicating her needs. Charlie cooks for them every day. Alina is dependent on Charlie's help during meals. He also has to cut her food into small pieces and pour the drinks for her.**

As an expert, how important do you think the following topics are for Charlie?

| | not important | | | | important |
|---|---|---|---|---|---|
| 1. Drinking – when, how much and what? | ○ | ○ | ○ | ○ | ○ |
| 2. Dealing with refusal to eat | ○ | ○ | ○ | ○ | ○ |
| 3. Ways to stimulate the appetite | ○ | ○ | ○ | ○ | ○ |
| 4. How does the sense of taste change with age? | ○ | ○ | ○ | ○ | ○ |
| 5. Suggestion (optional): | ○ | ○ | ○ | ○ | ○ |
| 6. Suggestion (optional): | ○ | ○ | ○ | ○ | ○ |
| 7. Suggestion (optional): | ○ | ○ | ○ | ○ | ○ |

Back Next

Leave and delete my data

Study coordinator Dominik Wolff Hannover Medical School PLRI Carl-Neuberg-Str. 1 D-30625 Hannover, Germany
Dominik Wolff – 2019

64% completed

**Anton and Marie**

**Anton is cared for by his 40-year-old daughter Marie, as he needs help with basic everyday tasks. Anton is demented, often disoriented and struggles with multi-step actions. Additionally, he has difficulties communicating his needs. Marie supports him with his meals.**

As an expert, how important do you think the following topics are for Marie?

| | not important | | | | important |
|---|---|---|---|---|---|
| 1. Dealing with refusal to eat | ○ | ○ | ○ | ○ | ○ |
| 2. Drinking – when, how much and what? | ○ | ○ | ○ | ○ | ○ |
| 3. Ways to stimulate the appetite | ○ | ○ | ○ | ○ | ○ |
| 4. Kitchen aids for demented persons | ○ | ○ | ○ | ○ | ○ |
| 5. Dealing with medication refusal | ○ | ○ | ○ | ○ | ○ |
| 6. Special characteristics of cooking for dementia sufferers | ○ | ○ | ○ | ○ | ○ |
| 7. How does the sense of taste change with age? | ○ | ○ | ○ | ○ | ○ |
| 8. Bladder workout | ○ | ○ | ○ | ○ | ○ |
| 9. Biography work used correctly | ○ | ○ | ○ | ○ | ○ |
| 10. Suggestion (optional): | ○ | ○ | ○ | ○ | ○ |
| 11. Suggestion (optional): | ○ | ○ | ○ | ○ | ○ |
| 12. Suggestion (optional): | ○ | ○ | ○ | ○ | ○ |

Back Next

Leave and delete my data

73% completed

**Dieter and Helga**

**55-year-old Dieter cares for his mother Helga. Helga needs assistance with everyday tasks such as washing, positioning, dressing and undressing. She is able to take meals herself, yet has become urinary incontinent and has been increasingly dependent on help in the last six months. Dieter wishes for support.**

As an expert, how important do you think the following topics are for Dieter?

| | not important | | | | important |
|---|---|---|---|---|---|
| 1. Assistive devices – everyday relief | ○ | ○ | ○ | ○ | ○ |
| 2. Tips for washing demented persons | ○ | ○ | ○ | ○ | ○ |
| 3. Possible constructional changes | ○ | ○ | ○ | ○ | ○ |
| 4. Fall prevention | ○ | ○ | ○ | ○ | ○ |
| 5. How can I help in case of a fall? | ○ | ○ | ○ | ○ | ○ |
| 6. Suggestion (optional): | ○ | ○ | ○ | ○ | ○ |
| 7. Suggestion (optional): | ○ | ○ | ○ | ○ | ○ |
| 8. Suggestion (optional): | ○ | ○ | ○ | ○ | ○ |

Back Next

Leave and delete my data

Study coordinator Dominik Wolff Hannover Medical School PLRI Carl-Neuberg-Str. 1 D-30625 Hannover, Germany
Dominik Wolff – 2019

82% completed

**Thomas and Stephanie**

**Thomas assists his demented mother Stephanie with basic activities such as personal hygiene. She is fecal incontinent and needs help with the toilet. Thomas often changes her incontinence materials, since Stephanie is not always able to communicate her needs and does not understand requests clearly. The fact that his mother is incontinent fills him with shame.**

As an expert, how important do you think the following topics are for Thomas?

| | not important | | | | important |
|---|---|---|---|---|---|
| 1. Dementia and fecal incontinence | ○ | ○ | ○ | ○ | ○ |
| 2. What is fecal incontinence? | ○ | ○ | ○ | ○ | ○ |
| 3. Dealing with refusal to eat | ○ | ○ | ○ | ○ | ○ |
| 4. Environmental design for dementia-related incontinence | ○ | ○ | ○ | ○ | ○ |
| 5. Bladder workout | ○ | ○ | ○ | ○ | ○ |
| 6. Environmental design for incontinence | ○ | ○ | ○ | ○ | ○ |
| 7. Handling incontinence material | ○ | ○ | ○ | ○ | ○ |
| 8. What is urinary incontinence? | ○ | ○ | ○ | ○ | ○ |
| 9. Clothing as an expression of individuality in people suffering from dementia | ○ | ○ | ○ | ○ | ○ |
| 10. Structuring the toilet calls | ○ | ○ | ○ | ○ | ○ |
| 11. What is a tendency to run? | ○ | ○ | ○ | ○ | ○ |
| 12. Wandering of demented persons | ○ | ○ | ○ | ○ | ○ |
| 13. Biography work used correctly | ○ | ○ | ○ | ○ | ○ |
| 14. How does the sense of taste change with age? | ○ | ○ | ○ | ○ | ○ |
| 15. Suggestion (optional): | ○ | ○ | ○ | ○ | ○ |

16. Suggestion (optional):

17. Suggestion (optional):

Back Next

Leave and delete my data

Study coordinator Dominik Wolff Hannover Medical School PLRI Carl-Neuberg-Str. 1 D-30625 Hannover, Germany
Dominik Wolff – 2019

91% completed

☐ I am interested in **the results of this study**. Please send me an abstract by e-mail.

Back

Next

Leave and delete my data

Study coordinator Dominik Wolff Hannover Medical School PLRI Carl-Neuberg-Str. 1 D-30625 Hannover, Germany
Dominik Wolff – 2019

# Thank you for completing this questionnaire!

We would like to thank you very much for helping us.

Your answers were transmitted, you may close the browser window or tab now.

Study coordinator Dominik Wolff Hannover Medical School PLRI Carl-Neuberg-Str. 1 D-30625 Hannover, Germany
Dominik Wolff – 2019
